# An overview of clinical presentation and management of long COVID

**DOI:** 10.1097/01.NPR.0000000000000374

**Published:** 2025-10-28

**Authors:** Sujith Sujith, Noah Gatzke

**Affiliations:** **Sujith Sujith** is a recent graduate of the NP program at the College of Nursing, Rady Faculty of Health Science at the University of Manitoba in Winnipeg, Manitoba, Canada. He currently works in urgent care in the WRHA.; **Noah Gatzke** is faculty in the College of Nursing, Rady Faculty of Health Science at the University of Manitoba in Winnipeg, Manitoba, Canada.

**Keywords:** COVID-19, health practitioners, long COVID, management, postacute sequelae of SARS-CoV-2 infection, postacute COVID syndrome, risk factor, SARS-CoV-2, vaccine

## Abstract

The COVID-19 pandemic has been the 21st century's most significant public health emergency. In addition to the acute symptoms of COVID-19, many individuals are facing long-term health issues related to the infection. The terms “long COVID,” “postacute sequelae of SARS-CoV-2 infection,” “postacute COVID syndrome,” and “long-haul COVID-19” refer to long-term consequences of SARS-CoV-2 infection. Symptoms may persist for weeks or months, reducing quality of life. Health practitioners must stay updated and take proactive measures to manage long COVID effectively. This manuscript provides an overview of risk factors, diagnostic tools, and management strategies, which serve as a resource for understanding and managing long COVID.

Around 10% of people who contract severe acute respiratory syndrome coronavirus 2 (SARS-CoV-2) infection, or COVID-19, experience incomplete recovery and severe, long-term health complications. This long-lasting, complex, and debilitating condition, which can affect multiple body systems, is called long COVID.^1^ Long COVID, also known as postacute COVID-19 sequelae, postacute sequelae of SARS-CoV-2 infection, long-haul COVID-19, postacute COVID syndrome, or post-COVID-19 syndrome, can persist for years and may pose a risk of long-term complications and multiorgan damage.^2^ The National Institute for Health and Care Excellence (NICE) defines the condition as “signs and symptoms that develop during or after an infection consistent with COVID-19, continue for more than 12 weeks and are not explained by an alternative diagnosis.”^3^ It's estimated that there are over 65 million cases of long COVID worldwide, with the number of reported cases increasing daily.^1^ This review aims to enhance understanding of long COVID and highlight potential treatment options in adults.

## RISK FACTORS

Long COVID can manifest in individuals across different age groups and varying degrees of acute COVID-19 severity. Several risk factors have been identified, including female sex; Hispanic or Latino ethnicity; socioeconomic disadvantage; current tobacco use; obesity; preexisting comorbidities including anxiety, depression, asthma, chronic obstructive pulmonary disease, diabetes, ischemic heart disease, and immunosuppression; and lack of COVID-19 vaccination.^1^,^4^ Of note, oral antiviral agents such as nirmatrelvir-ritonavir (Paxlovid) may help reduce the risk of developing long COVID, if started within 5 days of COVID-19 symptom onset.^5^

## PATHOPHYSIOLOGY

The infectious agent that causes COVID-19 is the single-stranded RNA coronavirus known as SARS-CoV-2.^6^ The virus enters the host through the respiratory tract and binds its spike protein to the angiotensin-converting enzyme II (ACE2) receptor to invade the host cell and start replicating.^2^ When the viral load is at its peak, symptoms begin to develop, ranging from a mild flu-like illness to acute respiratory distress syndrome and septic shock.^2^ The acute COVID-19 stage refers to the first 5 weeks after the initial infection. During this time, the viral load declines and becomes undetectable; the postacute stage then begins.

There are various overlapping hypotheses about the pathogenesis of long COVID. Persisting reservoirs of virus in tissues, microvascular blood clotting, endothelial dysfunction, elevated levels of autoantibodies, improper signaling of the brainstem and vagus nerve, and reactivation of dormant pathogens like Epstein-Barr virus and other herpes viruses are considered potential causes of long COVID.^1^

## SYMPTOMS

The primary initial symptoms of long COVID typically include fatigue, cognitive impairment, myalgia, arthralgia, palpitations, chest pain, dyspnea, anxiety, and depression.^2^ Patients can experience adverse effects across various organ systems, including neurological, cardiovascular, respiratory, gastrointestinal, reproductive, and immune systems, lasting years.^1^ Impacts of long COVID are described below.

### Neurological, psychiatric, and ocular

Neurological and cognitive symptoms comprise a significant aspect of long COVID. Possible symptoms include sensorimotor issues, memory loss, cognitive impairment (brain fog), paresthesia, dizziness, balance problems, sensitivity to light and noise, anosmia, ageusia, and autonomic dysfunction, which negatively impact activities of daily living.^7^ Cognitive impairment is often debilitating, may progress over time, and may be a direct effect of neuropathologies potentially involved in long COVID, like neuroinflammation, damage to blood vessels due to coagulopathy and endothelial dysfunction, and injury to neurons.^7^ Furthermore, the neuroinflammation and neuronal injury of long COVID could trigger the development of neurodegenerative diseases such as Alzheimer's or Parkinson's disease.^7^ SARS-CoV-2 can impact the permeability of the blood-brain barrier, causing cellular dysregulation and inflammation in the brain parenchyma.^7^

Postural orthostatic tachycardia syndrome (POTS), a form of dysautonomia, is a possible manifestation of long COVID and may be caused by inflammation of the autonomic nervous system.^8^

Studies have found that central nervous system disturbance occurs secondary to neuroinflammation and can result in neuropsychiatric abnormalities, including chronic malaise, fatigue, sleep disorders, ageusia, anosmia, posttraumatic stress disorder, anxiety, depression, and irritability.^2^,^9^

Corneal small nerve fiber loss and increased dendritic cell density have been seen in the eyes of patients with long COVID, as well as significantly impaired retinal microcirculation. Other ocular manifestations of long COVID include retinal hemorrhages, cotton wool spots, and retinal vein occlusion.^1^,^10^

### Cardiovascular

Cardiovascular symptoms of long COVID can include chest pain and palpitations.^8^ Cardiomyocytes have many ACE2 receptors, which provide direct access to SARS-CoV-2 infection, potentially leading to chronic inflammation, myositis, and cell death.^2^ Cellular damage and chronic inflammation can lead to arrhythmias and coagulopathy. Elevated serum troponin levels may also be seen in some patients with long COVID.^2^

### Respiratory

Studies show that dyspnea and cough are the most common respiratory symptoms reported with long COVID and are associated with a decreased quality of life.^6^ Several factors involved in developing dyspnea in long COVID include parenchymal sequelae, dysfunctional breathing, cardiovascular dysfunction, and muscular deconditioning.^6^ Long-term inflammation and viral-induced activation of the complement system disrupt coagulation pathways, leading to a hypercoagulable state that increases the risk of thrombosis.^11^ Research has shown that higher frequencies of SARS-CoV-2-specific T cells are associated with systemic inflammation, prolonged dyspnea, and reduced lung function, resulting in persistent pulmonary symptoms in long COVID.^12^ Some individuals may develop post-COVID-19 pulmonary fibrosis, which involves irreversible lung damage, including interstitial reticulation, irregular pleural interfaces, traction bronchiectasis, and honeycomb lesions. This condition is characterized by abnormal reconstruction of the alveolar epithelium, leading to excessive collagen production in the lungs and destruction of the normal lung structure.^6^

### Gastrointestinal

In long COVID, gastrointestinal signs and symptoms may include loss of appetite, nausea, weight loss, abdominal pain, heartburn, dysphagia, changes in bowel movements, and irritable bowel syndrome.^13^ These symptoms may be related to imbalances in gut bacteria, abnormal interactions between the nervous system and immune system, presence of ACE2 receptors in the esophagus, and unusual immune responses in the gut.^13^ These abnormal immune responses could be caused by the continued presence of the virus, ongoing inflammatory reactions, autoimmune reactions due to similarities between viral and human proteins, or impaired repair mechanisms in the gut.^13^

### Reproductive

Maintaining vascular integrity is essential for penile erectile function, but COVID-19 can cause damage to the endothelium, which can impair the penile vascular flow and lead to erectile dysfunction (ED).^14^ In addition, the virus can persist within the cavernosal endothelium, which may also contribute to the worsening of ED.^14^ Recent studies have shown that individuals with long COVID may have impaired semen quality, including decreased semen volume and decreased count, motility, morphology, and concentration of sperm. These impairments have been linked to elevated levels of cytokines in seminal fluid.^15^

Menstrual changes like irregular or infrequent menstruation and increased premenstrual syndrome may occur in long COVID.^16^ Reduced ovarian reserve and improper endometrial response may be seen in long COVID due to the abundance of ACE2 receptors on ovarian and endometrial tissue.^16^

### Immune

Studies have found immune dysregulation in long COVID with specific T cell changes, including exhausted T cells, reduced numbers of effector memory cells (CD4+ and CD8+), and increased PD-1 (programmed cell death protein 1) expression on central memory cells, that may persist for at least 13 months.^17^ Furthermore, elevated cytokine levels and high levels of autoantibodies in long COVID suggest that patients are more susceptible to multisystem injury.^6^

## DIAGNOSIS

A diagnosis of long COVID can be considered for patients with signs or symptoms developing during or after COVID-19 (or an infection consistent with COVID-19) and persisting for longer than 12 weeks, once alternative diagnoses for the signs and symptoms have been excluded.

For patients with suspected long COVID, laboratory tests that providers may consider ordering include complete blood cell count, kidney and liver function tests, C-reactive protein (CRP), ferritin, B-type natriuretic peptide, and thyroid function tests.^3^ Elevated systemic inflammatory markers like D-dimer, CRP, interleukin-6, procalcitonin, and neutrophil count are associated with prolonged symptoms of long COVID.^2^,^18^

Clinicians should assess exercise tolerance with a 1-minute sit-to-stand test, measuring breathlessness, heart rate, and oxygen saturation.^3^ Pulmonary function tests (PFTs) should be obtained for patients with respiratory symptoms that have not improved within 8 weeks of symptom onset; PFTs can be considered earlier for patients with worsening symptoms.^19^ Chest X-ray is recommended if the person has dyspnea or other ongoing respiratory symptoms.^3^ A chest CT should also be obtained for patients with persistent radiographic abnormalities and progressive respiratory symptoms.^19^,^20^

In patients reporting weakness or fatigue, it is recommended that clinicians perform a 12-lead electrocardiogram and obtain laboratory testing, including cardiac troponin and D-dimer.^21^ For patients with a history or biochemical evidence of myocardial injury or myocarditis and signs or symptoms suggestive of an underlying cardiac disorder, transthoracic echocardiography should be obtained.^21^ For patients with palpitations or symptoms of dysautonomia, a 10-minute active stand test should be performed with measurement of heart rate and blood pressure 5 minutes after lying supine, immediately upon standing, and at 2, 5, and 10 minutes of standing to evaluate for orthostatic hypotension and POTS. In POTS, typically the heart rate after standing for 5-10 minutes is more than 30 beats per minute faster than the supine heart rate and orthostatic hypotension is absent.^21^ If POTS is suspected and/or patients develop significant symptoms while standing, tilt table testing can be used for further assessment. Holter monitoring can also be considered.^21^

Diagnostic tools for long COVID are still being developed, including imaging techniques that can detect microclots, corneal microscopy that can identify small fiber neuropathy, and the use of hyperpolarized MRI to detect abnormalities in pulmonary gas exchange.^1^,^22^,^23^ More research in developing and validating biomarkers in long COVID will help diagnose long COVID and define treatment responses.^1^

## MANAGEMENT

Medical experts are making significant efforts to manage patients with long COVID. Although several guidelines for long COVID management have been issued, there is still a substantial practical gap, and specific treatments have not been thoroughly evaluated. As empirical evidence is yet to emerge to inform clinical practice, a comprehensive review of the clinical strategies used by healthcare providers will be valuable in guiding effective patient rehabilitation.

### Neurological and psychiatric

For patients with POTS, nonpharmacological therapy, including gradual exercise regimens, increased fluids and salt intake, compression stockings, abdominal binders, counter maneuvers such as leg crossing, and behavioral modifications, is first-line treatment.^1^ Pharmacological therapy is reserved for patients who do not fully respond to supportive care; though not FDA-approved for POTS, some medications, such as beta-blockers and fludrocortisone, are prescribed off-label for this indication.^1^

Two patients with long COVID in a case series experienced significant alleviation of dysautonomia symptoms after undergoing stellate ganglion block.^24^

For individuals who exhibit severe psychiatric symptoms or are at risk of self-harm or suicide, an urgent psychiatric assessment is necessary.^3^ Low-dose naltrexone can potentially prevent and treat immune-mediated thrombotic complications and neuroinflammation in long COVID.^25^ Antihistamines like famotidine may help alleviate neuropsychiatric symptoms and brain fog.^26^ Management of anxiety and depression should follow standard guideline recommendations.^3^

### Cardiovascular

A cardiac consultation should be obtained for patients with abnormal cardiac test results and/or a history of cardiac complications during infection with COVID-19.^21^

Anticoagulant regimens have shown positive results in managing abnormal clotting and may need to be considered for certain patients.^27^

### Respiratory

Patients with mild dyspnea should be prescribed breathing exercises (eg, diaphragmatic breathing), chest physiotherapy, and breathlessness management strategies through self-directed educational resources, in-person rehabilitation, or online programs.^19^ Patients with dyspnea are often prescribed inhalers, such as short-acting beta-adrenergic and inhaled corticosteroids, or oral glucocorticoids.^19^ A trial of these medications for a short duration of time on an individual basis can be considered; however, routine use of pharmacotherapies for dyspnea is not recommended, unless supported by clinical assessment and testing.^19^ Patients who experience moderate to severe dyspnea or persistent drops in oxygen level or who consistently require oxygen therapy should be referred to a pulmonary specialist for further investigation and for pulmonary rehabilitation.^19^

### Gastrointestinal

Pilot studies involving the use of probiotics have also been carried out, indicating their potential to alleviate gastrointestinal and nongastrointestinal symptoms.^28^,^29^

## VACCINATION

The effect of COVID-19 vaccination on the occurrence of long COVID varies among studies. Many studies indicate that COVID-19 vaccination is associated with reduced risk of long COVID.^19^,^30^ However, some studies suggest no significant difference in the development of long COVID between vaccinated and unvaccinated individuals.^5^,^31^,^32^

## REHABILITATION

Referring individuals with long COVID to multidisciplinary assessment and rehabilitation services, including physical and occupational therapy, pulmonary or cardiac rehabilitation, and speech and swallowing therapy, is recommended once life-threatening complications and alternative diagnoses have been ruled out.^3^ All patients should be screened for cardiac symptoms before beginning any exercise program; if necessary, a complete cardiac evaluation may be warranted before commencing rehabilitation therapy based on symptoms.^21^

## NP PRACTICE IMPLICATIONS

Long COVID requires multidisciplinary management and is a significant concern for nurse practitioners. The long-term complications of COVID-19 may lead to a substantial increase in the use of health resources. Nurse practitioners must be aware of the potential mechanisms causing persistent symptoms in these patients in order to develop individual management strategies tailored to each patient's condition. The management of chronic complications from long COVID will require a multidisciplinary approach, which will be a significant focus for nurse practitioners in the future.

## CONCLUSION

Long COVID is a complex illness affecting multiple organ systems. It has impacted millions worldwide and will continue challenging the health care system and the economy. More research is needed to improve patient outcomes, prioritize health care resources, and improve training for health care providers. Practitioners should strive to stay updated on current management approaches so that they can take proactive measures to manage long COVID effectively.
